# Comment on ‘Effect of Hyperthermic Intraperitoneal Chemotherapy (HIPEC) on survival and recurrence rates in advanced gastric cancer – a systematic review and meta-analysis’

**DOI:** 10.1097/JS9.0000000000000679

**Published:** 2023-09-02

**Authors:** Xian-Wen Liang, Hui Liu, Jin-Cai Wu

**Affiliations:** a Department of Hepatobiliary and Pancreatic Surgery, Hainan General Hospital (Hainan Affiliated Hospital of Hainan Medical University); b Department of Interventional Radiology, Central South University, Xiangya School of Medicine Affiliated Haikou Hospital, Haikou, Hainan Province, People’s Republic of China

*Dear Editor*,

We read with great interest the recent article titled ‘Effect of Hyperthermic Intraperitoneal Chemotherapy (HIPEC) on survival and recurrence rates in advanced gastric cancer – a systematic review and meta-analysis’^1^ published in *International Journal of Surgery*. Based on 16 studies [6 randomized controlled trials (RCTs) and 10 observational studies], including a total of 1700 participants, the authors concluded that in patients with locally advanced gastric cancer with macroscopic serosa-invasion as well as peritoneal carcinomatosis that underwent resection with curative intent, combination with HIPEC could significantly improve overall survival rates and reduce recurrence rates without a significant increase in complications. We commend the authors for their efforts in addressing this important clinical issue. Nevertheless, several issues needed to be clarified to further refine this significant study.

First, according to the inclusion criteria of this study, HIPEC was defined as an intervention. However, when we carefully checked the included studies, we found that an RCT (with 268 patients) was conducted by Miyashiro *et al*.^2^ contradicted the inclusion criteria, which merely used intraperitoneal chemotherapy without hyperthermic perfusion as an intervention. From a rigorous perspective, this article should not be included in the meta-analysis.

Second, a comprehensive literature search is an important prerequisite for a reliable meta-analysis. In this study, only PubMed, EMBASE, MEDLINE, and Cochrane databases were searched. Other databases, including the Web of Science and Google Scholar, should also be searched. This meta-analysis did not include the studies by Geng *et al*.^3^, Bonnot *et al*.^4^, and Yin *et al*.^5^. By careful evaluation of the full text, we found that these studies met the inclusion criteria for this meta-analysis, and the authors should have included them in the present study for analysis.

Third, we found that the authors did not assess the risk of bias in the included studies accurately. For example, in the included RCTs, due to the nature of the study intervention, blinding of investigators and participants was not possible, so all studies in this domain should be high risk. In addition, none of the RCTs mentioned the details of blinding of outcome measurement; thus, the risk of outcome measurement should be unknown in these studies.

Finally, testing for publication bias for the primary and secondary outcomes was missing in the present study. Significant publication bias has a serious impact on the reliability of the pooled results. According to Cochrane’s guidelines, Begg’s and Egger’s tests are recommended to clarify this concern.

Overall, we thank Patel and colleagues from the bottom of our hearts for their concerted efforts in investigating the therapeutic efficiency of HIPEC in advanced gastric cancer. They have made an important step forward on this clinically meaningful topic. But we have just pointed out our concerns, which might help the authors make their findings clearer.

## Ethical approval

There is no need for this Letter to the Editor.

## Sources of funding

None.

## Author contribution

L.X.-W. and L.H.: original draft conception and writing; W.J.-C.: critical revision of the manuscript. All authors reviewed the manuscript.

## Conflicts of interest disclosure

The authors declare that they have no conflicts of interest.

## Research registration unique identifying number (UIN)

1. Name of the registry: none.
2. Unique identifying number or registration ID: none.
3. Hyperlink to your specific registration (must be publicly accessible and will be checked): none.

## Guarantor

Wu Jin-Cai.

## Provenance and peer review

Commentary, internally reviewed.
